# Survival Analysis in Patients with Chronic Traumatic Spinal Cord Injury

**Published:** 2019-12

**Authors:** Mahsa GHAJARZADEH, Abbas RAHIMI FOROUSHANI, Saharnaz NEDJAT, Abdolreza SHEIKHREZAEI, Hooshang SABERI

**Affiliations:** 1. Brain and Spinal Cord Injury Research Center, Tehran University of Medical Sciences, Tehran, Iran; 2. Department of Epidemiology and Biostatistics, School of Public Health, Tehran University of Medical Sciences, Tehran, Iran; 3. Department of Epidemiology and Biostatistics, School of Public Health, Knowledge Utilization Research Center, Tehran University of Medical Sciences, Tehran, Iran; 4. Department of Neurosurgery, School of Medicine, Tehran University of Medical Sciences, Tehran, Iran

**Keywords:** Survival, Traumatic spinal cord injury, Mortality, Iran

## Abstract

**Background:**

The goal of this study was to determine hazard rate of death rate and the causes of death in Iranian patients with Traumatic spinal cord injury (TSCI).

**Methods:**

Overall, 1024 patients with chronic traumatic spinal cord injury referred to Brain and Spinal Injury Research Center, Tehran University of Medical Sciences, Tehran, Iran from Jan 2013–2017 were enrolled. Epidemiological and neurological data, along with secondary complications were recorded for all participants. In the case of death, the cause, and the date of death were recorded. The Kaplan–Meier method was used for survival analysis. A log-rank test was carried out to compare survival due to different risk factors. Risk factors and relative risk estimates associated with death were assessed by means of a Cox regression model.

**Results:**

Nineteen percent were lost to follow up. During the follow-up period, 22 out of 830 remaining cases (2.6%) died. Deaths were only observed in patients with cervical injuries (59% in C1–C4 level and 41% in C5–C7 level). Kaplan–Meier Log-rank test showed that probability of survival was significantly less in females, complete injury cases, patients with cervical spine injury, depression, and ADR (Autonomic dysreflexia). Controlling for age, sex and education level, Cox regression model showed that hazard rate of death was significantly affected by the categorical variables such as level of injury (HR=0.2, 95% CI=0.12–0.39), severe ADR.

**Conclusion:**

Probability of survival is lower in female individuals, cases with complete injuries, patients with cervical spine injury, individuals with depression (BDI>10), and clients who experience ADR.

## Introduction

Traumatic spinal cord injury (TSCI) is a catastrophic event affecting all aspects of patient's life (1). It is associated with higher rates of mortality and morbidity. In recent decades, survival rate in patients with TSCI has increased dramatically due to improved medical care such as acute phase care, early post-injury management, and post-acute rehabilitation modalities (2–5). The risk of death is greater during the first two years after the injury (6–8)^.^ Mortality in patients with TSCI has been reported to be three times more than that in age-matched healthy subjects (9).

Demographic and neurological factors such as age at the time of injury, sex, and neurological level and severity were considered as major predictors of survival, although there has been discrepancy between results of different studies worldwide (9–12). Previously, urinary complications were the most common causes of death in the chronic phase, while recently cardiovascular events and respiratory failure, suicide, and septicemia have become the most common causes of death after TSCI (13–15). More national studies evaluating survival, risk factors and the causes of death in TSCI cases are required to help health systems to set up primary and secondary prevention strategies, care policies, and financial programming and support.

Using the related findings from other countries is not recommended due to different population characteristics, statistical methods, level of care, and financial status among various societies. As to our best knowledge, there is no similar study in Iran, we designed this study to evaluate the mortality rate, risk factors and causes of death in Iranian subjects with chronic TSCI.

## Material and Methods

This prospective cohort study was conducted in Brain and Spinal Injury Research Center (BASIR) (Tehran University of Medical Sciences) between Jan 2013 and 21^st^ May 2017. Case recruitment was done between Jan 2013 till Jan 2014 then all enrolled cases followed up till May 2017 to register death event.

Inclusion criteria were: Traumatic etiology for spinal cord injury, duration of spinal injury more than one year (prevalent cases), and predominant disability due to TSCI in cases with concomitant brain injury. Exclusion criteria were: unwillingness to participate in the study, and inaccessibility due to address change. From 1600 registered cases in BASIR center followed up for at least one year after injury (for cases referred at the time of injury or in acute phase), 1024 eligible cases (who had inclusion criteria) were considered. Among them, 194 were lost to follow up and finally, 830 cases completed the study (Response rate=81%).

All participants signed the filled informed consent forms before the study.

The study had been approved by local Ethical Committee (ID: 25661).

Data regarding age, sex, injury date, education level, marital status, and mechanism of injury were recorded for all participants. Each patient was examined by the attending neurosurgeon and the SCI research fellow. After a comprehensive neurological examination, all the possible complications including pressure ulcer (PU), neuropathic pain (NP), autonomic dysreflexia (ADR), urinary tract infection (UTI), heterotopic ossification (HO), spasticity(SP), urinary tract calculi (UTC), pneumonia, suicidal attempt, sexual dysfunction, and depression were assessed and recorded. Spinal Cord Independence Measure (SCIM) questionnaire was filled out for all patients during the follow-up period. SCIM contains 19 items assessing three Domains: self-care, respiration and sphincter control, and mobility, with a total score ranging from 0 to 100.

Patients who missed their appointments during the follow-up period and those who were not willing to continue participating in the study were considered as censored cases. Considering the age at the time of injury, patients were divided into three groups: Less than 30 yr, 30–59 yr, and more than 59 yr old (5). There were two educational level groups: ≤12 yr, >12 yr (16). The level of injury was categorized as upper cervical (C1–C4), lower cervical (C5–C7), upper thoracic (T1–T6), lower thoracic (T7–T12), or lumbar (L1–L5) (17).

### Statistical analysis

Analysis was conducted by means of STATA software version 14 (StataCorp, College Station, TX, USA). Chi-square test was applied for comparison of categorical variables. Independent sample t-test was used to compare continuous variables. The Kaplan–Meier method was applied for survival analysis, obtaining survival curves. Log-rank test was carried out to compare survival rate between various groups with different risk factors. Risk indicators and relative risk estimates associated with mortality were assessed by the Cox regression model. Cox proportional hazard models were used to assess the effects of potential risk factors. The Hazard ratio (HR) and 95% confidence interval (CI) were calculated. Considering age, sex, education level as confounders, different models were assessed. Cases, lost to follow up, were compared with deceased cases regarding age, sex, as well as level and severity of the injury. A *P*-value less than 0.05 was considered to be statistically significant.

## Results

During the follow-up period, 22 out of 830 cases (2.6%) died. Four hundred ninety one cases (61%) were under 30 yr and 81% had equal or less than 12 yr of education. Male to female ratio was 4.3. The median time since injury till 21^st^ of May 2017 was 8.5 years. Mean total SCIM score was 52.6. The most common site of injury was at the lower thoracic spine (Table 1).

**Table 1:** Demographic data of patients (N=1024)

***Variables***	***Total***
Age at examination (mean ±SD) (yr)	29±11.9
Age at the time of the injury	
<30	491(61%)
30–59	308(38 %)
≥60	31(1%)
Education years	
≤12 years	676(81%)
>12 yr	154(19%)
Sex	
Male	674(81%)
Female	156(19%)
M/F=4.3	
Marital status	
Single	325(39%)
Married	479(58%)
Divorced	24(2.8%)
Widowed	2(0.2%)
Occupational status	
Employed	133(16%)
Unemployed	652(79%)
Lost-job	12(1%)
Student	33(4%)
Level of injury	
C1–C4	43(5%)
C5–C7	60(7)
T1–T6	162(19.5%)
T7–T12	435(52.4%)
L1–L5	130(16%)
Neurological status (AIS)	
A	537(65%)
B	126(15%)
C	131(16%)
D	36(4. %)
Etiology	
Vehicle accidents	501(60%)
Falling	249(30%)
Others	80(10%)

Comparison of lost to follow up patients and deceased cases by means of independent t-test and Chi-square tests showed that they were not different regarding age (*P*=0.1), sex (*P*=0.06), level of injury (*P*=1) and AIS (*P*=0.6).

Examination of patients showed that spasticity and depression (BDI ≥10) were the most common associated complications in our cases (63% and 47%, respectively) and heterotopic ossification (3%) besides pneumonia (2%) were the least common complications (Table 2).

**Table 2:** Frequency of various complications in 830 clients

***Complications***	***Frequency (%)***
Pressure ulcer	221(27)
Neuropathic pain	367(44)
Autonomic dysreflexia (ADR)	101(12)
Urinary tract infection	319(38)
Heterotopic ossification	26(3)
Spasticity	521(63)
Urinary tract calculi	103(12)
Pneumonia	20(2)
Suicide attempt	46(5.5)
Depression	389(47)
Sexual dysfunction	197(24)

In all 22 deceased cases, injury level was at cervical spine; thirteen in upper cervical (59%) (C1–C4) and 9 (41%) in lower cervical level (C5–C7). Eleven patients died at home (50%) and 11 in the hospital settings. The duration of injury, SCIM, sex, injury level, AIS, and mood status (BDI ≥10) were significantly different between deceased (before death), and survived cases (Table 3).

**Table 3:** Comparison of various factors between deceased and survived cases

**P-*value***	***Survivors***	***Deceased***	***Variable***
0.2	491(61%)	11(50%)	Age at the time of injury <30
	308(38%)	10(45.5%)	Age at the time of injury 30–59
	9(1%)	1(4.5%)	Age at the time of injury ≥60
0.007	98.8±61.9	62.9±31.2	Mean time since injury (months)
0.01	55.1±19.9	50±5	Mean SCIM (Spinal Cord Independence Measure)
0.03	148(18%)	8(36%)	Gender:Female
	660(82%)	14(64%)	Gender: Male
<0.001	30(4%)	13(59%)	Neurological level:C1–C4
	51(6%)	9(41%)	Neurological level: C5–C7
	162(20%)	0	Neurological level :T1–T6
	435(54%)	0	Neurological level :T7–T12
	130(16%)	0	Neurological level :L1–L5
0.008	527(65%)	10(45.5%)	AIS: A
0.008	117(15%)	9(41%)	AIS :B
0.008	129(16%)	2(9%)	AIS :C
0.008	35(4%)	1(4.5%)	D AIS:
<0.001	368(45%)	21(95%)	Mood status (depression) (BDI>=10)

The mechanism of the injury and educational level were not significantly different between deceased and survived cases. The most common cause of death was sepsis (13 cases died due to sepsis, 52%), and the most underlying cause of sepsis was PUs (6 cases, 46%), followed by UTI (2, 15%). In 5 cases (39%), the underlying cause of sepsis was unknown. The second cause of death was respiratory failure in 4 patients (18%) besides renal failure and GI (gastrointestinal) complications, both were the third causes of death (2 cases (18% died due to renal failure and 2 (18% due to GI complications).

Suicide was the cause of death in one individual (overdose of drugs) who’s BDI score was 30 (severely depressed)

Kaplan-Meier log-rank test showed that probability of survival was significantly lower in female individuals (K^2^ log rank=4.3, *P*=0.03), complete injuries (K^2^ log rank=8.8, *P*=0.03), cervical injuries (K^2^ log rank=71.4, *P*<0.001), depression (K^2^ log rank=24.2, *P*<0.001), or severe ADR (K^2^ log rank=6.5, *P*=0.01).

**Fig. 1:**
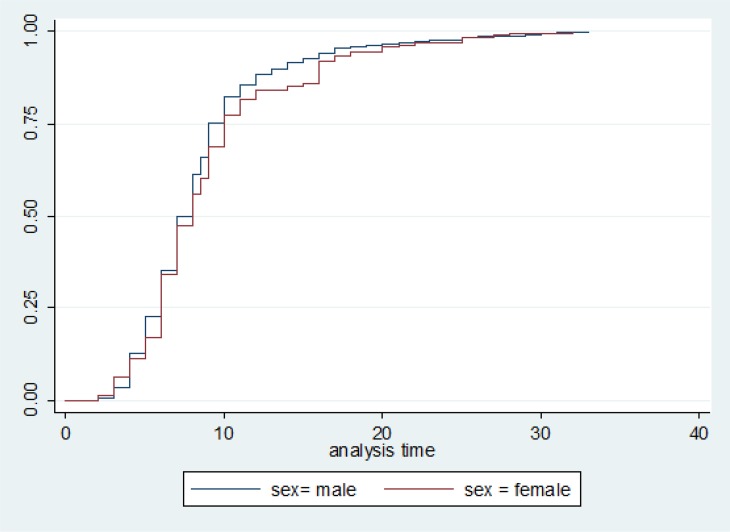
Hazard function of Traumatic Spinal Cord Injury by gender

**Fig. 2:**
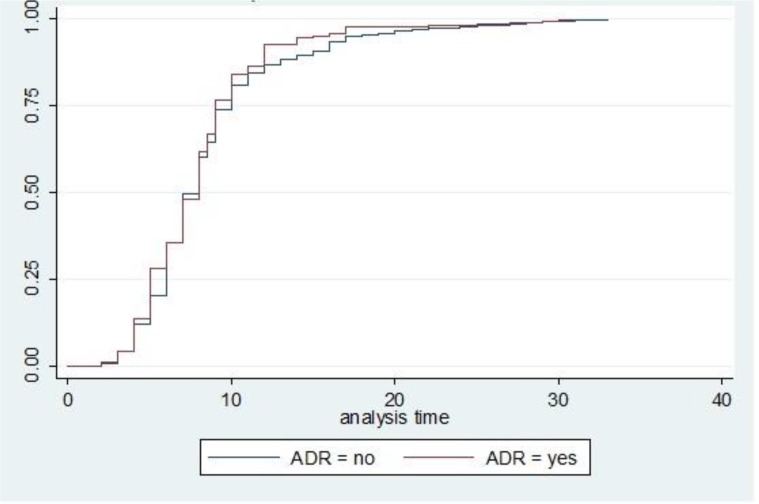
Hazard function of Traumatic Spinal Cord Injury by autonomic dysreflexia

Controlling for age, sex and education level, Cox regression model showed that hazard rate of death was significantly affected by level of injury (HR= 0.2, 95% CI=0.12–0.39), ADR (HR= 2.8, 95% CI=1.2–7.1) and BDI ≥10 (HR=5, 95% CI (1.8–14.1), (Fig. 1). Comparison of deceased and lost to follow up cases showed that they were similar regarding level and severity of injury, mean age and sex ratio(Fig. 2).

## Discussion

To our knowledge, this is the first study assessing survival rate of Iranian patients after chronic traumatic spinal cord injury, referred to a tertiary clinic. During the follow-up period, 22 cases died, and the most common cause of death was sepsis (59%). In a previous study, medical records of TSCI cases in Norway were reviewed and found cardiovascular events and suicide as the leading causes of death (2). Including patients with TSCI survived one-year post-injury, respiratory problems, cardiovascular events, and systemic neoplasms were reported as the leading causes of death in British TSCI patients(18). In Germany, the most frequent causes of death were septicemia, influenza/pneumonia, and suicide in TSCI cases with tetraplegia, and ischemic heart disease, neoplasms, and septicemia in those with paraplegia (19). In Denmark, urinary system complications, ischemic heart disease and respiratory complications were the most common causes of death among 169 SCI patients followed up for 25 years after the injury (20). Amongst Israeli SCI veterans who survived at least 10 years post-injury, genito-urinary complications and cardiovascular events were the most common causes of death (21). In this study, the most common complications were spasm, depression (BDI ≥10) and neuropathic pain.

In general, septicemia, originating from infections of the urinary tract, PU, or respiratory tract, is an issue of concern in SCI patients (22). In the current study, septicemia was the first cause of death, and the most common underlying cause of septicemia was pressure ulcer. As our results show, 31% of our cases had active PU.

PU are one of the most common secondary complications of spinal cord injury, which may be experienced by 85% of patients during lifetime (23). PU are more common in tetraplegics and their prevalence increases by disease chronicity (24–26). PU are life-threatening complications, and their incidence is considered as a major indicator for quality of care (27). Mortality in 230 Vietnam veterans (who survived beyond triage) within 25 years was analyzed and found sepsis as the cause of death in 38% of cases (28), and similar to our findings, the most common cause of sepsis was PU.

Previously, genitourinary complications were among major causes of death in SCI cases, while recently, this etiology is less likely because of tight evaluation, routine laboratory assessment, early diagnosis, and timely treatment (13). Nine percent of mortalities in our study was due to renal failure. Over 80% of SCI cases have abnormal lower urinary tract function. UTI, upper and lower urinary tract involvement and urolithiasis are prevalent in SCIs (29, 30). In the current study, 129 cases (13%) had urolithiasis while 38% had UTI.

Respiratory complications were also previously among the crucial causes of death, while due to proactive respiratory management and rehabilitation modalities such as chest-physiotherapy, respiratory-related death rate has decreased in recent years (22). We found respiratory complications as the second cause of death in our patients (18%), while in an Australian study, respiratory complications contributed to 36 out of 195 deaths in SCIs as the first death cause (18%) (22) in contrast to our findings.

Suicide was the cause of death for only one individual in this study, while it is among the common causes of death following SCI in Norwegian population (12). Overall, in patients with spinal cord injury, the rate of suicide attempts is 2–6 fold more common than in the general population (31, 32). Suicidal attempt was observed in 5% of our patients, which could be the consequence of major depression (BDI>29).

The prevalence of suicide in Iranian general population reported as 1% (33) which shows that suicidal attempt was near four-fold of general population.

The prevalence of depression (BDI≥10) in SCI individuals varies between 11%–78% (34–38). By means of BDI, we found that 47% were depressed (BDI≥10). In Norwagian SCI population, near 6% of deaths were due to suicide attempts (39). Suicide attempt was the cause of TSCI in 3% of British patients and 4% of deaths after TSCI were as the consequence of suicide (40).

Depression is among the most common psychological problems after SCI, and its prevalence in SCI patients is three-fold more than that in general population (41, 42). Increased hospital stay, decreased social integration, impaired quality of life, dependency in self-care, and lower patient activity are among consequences of depression in TSCI patients (43, 44). Using the Kaplan–Meier method, survival curves showed that depressed patients (BDI ≥10) had lower survival rate than non-depressed ones (BDI<10). Controlling for the confounding variables (age, sex, and education level), we found that mood status (BDI ≥10) was also a negative predictor of survival in this study (HR=5, 95% CI (1.8–14.1). The prevalence of depression varies in different studies due to different sample sizes, inclusion and exclusion criteria, and assessment instruments. Dryden et al reported depression in near one-third of Canadian TSCI cases (45), while in a study in Iran it was reported in near 49% of subjects (46).

Autonomic dysreflexia is a complication of SCI which could occur any time after injury, mostly occurring in patients with injury at T6 or above (47). It may be associated with myocardial ischemia and/or cerebral hemorrhage (48). Bladder and bowel distentions are the most common provokers of ADR. Upright position, removing tight clothes and tight control of blood pressure are necessary when an episode of ADR occurs (48). The Cox regression showed that the hazard ratio for severe ADR was 2.8, and the survival curves for the patients who experienced severe ADR, and those who did not, were significantly different.

In virtual studies, survival was strongly related to level and severity of injury (7, 9, 49). According to our results, all the deceased cases had the injury level at the cervical spine (mostly C1–C4), and the severity of injury was significantly associated with mortality. These findings are consistent with another findings (39). Patients with cervical injury need intensive care and rehabilitation programs, but lack of proper facilities for these cases in Iran leads to higher mortality rate.

Mortality in this study was significantly higher in women (18% of all cases were female while 36% of deceased cases were female). The odds ratio for sex was 2.5, (95% CI was 1.05–6.1). In contrast to our findings, in Denmark, patients mortality rate was not significantly different between male and female individuals (5), and it was higher among patients above 60 yr old. In current study, 1% of all cases were above 60 yr while mortality in cases more than 60 was 4.5%. This indicates that mortality was higher among elderly patients.

This study had some limitations. First, it was conducted in a tertiary hospital. Second, we had no information regarding cases that were not accessible. Multi-centric studies with efforts to cover all cases in the study period are recommended.

## Conclusion

Probability of survival is lower in female individuals, cases with complete injuries, patients with cervical spine injury, depressed individuals, and clients who experience ADR.

## Ethical considerations

Ethical issues (Including plagiarism, informed consent, misconduct, data fabrication and/or falsification, double publication and/or submission, redundancy, etc.) have been completely observed by the authors.
